# Qualitative and quantitative chemical analysis of *Leptadenia hastata*: exploring a traditional african medicinal plant

**DOI:** 10.3389/fchem.2024.1397549

**Published:** 2024-04-19

**Authors:** Jiangsheng Zhang, Yi Nan, Jie Su, Aminu Usman Jibril, Guiyuan Lv

**Affiliations:** ^1^ College of Pharmaceutical Sciences, Zhejiang Chinese Medical University, Hangzhou, China; ^2^ Graduate School, Tianjin University of Traditional Chinese Medicine, Tianjin, China; ^3^ Graduate School, Department of Computer Science, Bayero University, Kano, Nigeria

**Keywords:** *Leptadenia hastata*, quality control, flavonoid, UHPLC-Q-TOF/MS, UHPLC-DAD

## Abstract

*Leptadenia hastata* (Pers.) Decne is a commonly used food source and prescribed as a traditional African medicine for treatment of various diseases, such as diabetes, skin disorders, wounds, and ulcers. However, quality control has become a bottleneck restricting the therapeutic development and utilization of this plant. In this study, a reliable method for qualitative and quantitative determination of components in *Leptadenia hastata* was established. The components of *L. hastata* were profiled using ultra-high performance liquid chromatography coupled with quadruple time-of-flight tandem mass spectrometry (UHPLC-Q-TOF-MS). Subsequently, an ultra-high performance tandem diode array detector (UHPLC-DAD)-based method was used for simultaneous quantitative analysis of five major constituents in six batches of *L. hastata* samples. As a result, 35 compounds were tentatively identified. The quantities of the five constituents (vicenin-Ⅱ, orientin, schaftoside, chrysin 6-*C*-arabinoside 8-*C*-glucoside, chrysin 6-*C*-glucoside 8-*C*-arabinoside) were determined as 124.8–156.9 μg/g, 170.5–216.0 μg/g, 61.31–93.73 μg/g, 85.13–119.3 μg/g and 99.82–129.4 μg/g, respectively. This method offers a successful strategy for precise and effective evaluation of the constituents of *L. hastata*, providing a robust foundation for holistic quality assessment of medicinal plants.

## 1 Introduction

Indigenous plants in Africa play a vital role in supporting local populations since they are utilized as food, health products, and herbs (Aquino and Pizza, 1995; Mathieu and Meissa, 2007; Aliero and Wara, 2009). *Leptadenia hastata* (Pers.) Decne, a plant belonging to the Asclepiadaceae family, is widely distributed in tropical Africa (Aquino and Pizza, 1995; Aliero and Wara, 2009). Its flowers, leaves, and young shoots are used as a source of nutrition during times of famine due to their valuable nutrients (Freiberger et al., 1998; Sena et al., 1998). Additionally, the plant is renowned for its medicinal properties and traditionally used to treat various ailments, including diabetes, skin diseases, wounds, and ulcers. To our knowledge, no quality control studies have been reported for this essential African traditional herb to date.

Earlier studies have reported that the aqueous leaf extract of *L. hastata* has significant benefits in lowering fasting blood glucose levels, along with antidiabetic and hypoglycemic effects (Manosroi et al., 2011; Sanda et al., 2013; Oche et al., 2022). Acute systemic, topical and chronic anti-inflammatory properties of *L. hastata* leaf extracts have been demonstrated (Nikiéma et al., 2001; Ezike et al., 2016). Recent research has revealed a hepatoprotective effect of *L. hastata* (Ojochegbe et al., 2019; Galani et al., 2020). Moreover, *L. hastata* provides pharmacological and biological benefits, such as antimicrobial, anti-androgenic, antiprotozoal, and anti-invasive pulmonary aspergillosis activities (Bayala et al., 2011; Bello et al., 2017; Bello et al., 2019; Abdallah and Ali, 2022). Accordingly, establishing an effective quality control method for this herb is essential to maximize its pharmaceutical utilization.

Qualitative phytochemical screening analysis of *L. hastata* in previous studies revealed the presence of flavonoids, glycosides, saponins, tannins, triterpenes and alkaloids (Manosroi et al., 2011; Ezike et al., 2016; Chukwuma et al., 2022). To identify components of a stem aqueous decoction of *L. hastata*, high-performance liquid chromatography (HPLC) was utilized, which revealed the presence of quinine, scopoletin, and dihydroxycoumarin (Galani et al., 2020). A gas chromatography-mass spectrophotometer (GC-MS) and gas chromatography–flame ionization detector (GC-FID) were recently employed for qualitative analysis of the components of *L. hastata*, which led to the identification of compounds with weak polarity and volatility (Chukwuma et al., 2022). These compounds may be responsible for the observed pharmacologic activities, with flavonoids identified as one of the main active constituents of this plant (Ezike et al., 2016; Chukwuma et al., 2022). However, chemical profiling analysis is insufficient and quantitative analysis of flavonoids of *L. hastata* is lacking at present.

In this study, ultra-high performance liquid chromatography coupled with quadruple time-of-flight tandem mass spectrometry (UHPLC-Q-TOF/MS) was employed to evaluate the chemical characteristics of *L. hastata* for further clarifying the material basis of the medicinal effect. On this basis, an ultra-high performance tandem diode array detector (UHPLC-DAD) method for simultaneous and quantitative detection of five flavonoids, specifically, vicenin-Ⅱ, orientin, schaftoside, chrysin 6-*C*-arabinoside 8-*C*-glucoside, and chrysin 6-*C*-glucoside 8-*C*-arabinoside, was established for quality control of *L. hastata*.

## 2 Materials and methods

### 2.1 Plant materials

The leaves and shoots of *L. hastata* were gathered from their natural habitat in Kano State, Nigeria. Plants were identified and authenticated by Professor Bala Sidi Aliyu from the Department of Plant Biology and Professor Fatima Batul Mukhtar from the Department of Biological Sciences at Bayero University (Kano State, Nigeria). Six batches of *L. hastata* (lot numbers: 20211010, 20211022, 20211109, 20220107, 20220206 and 20220322) were collected from Kano, Nigeria.

### 2.2 Chemical and reagents

**FIGURE 1:**
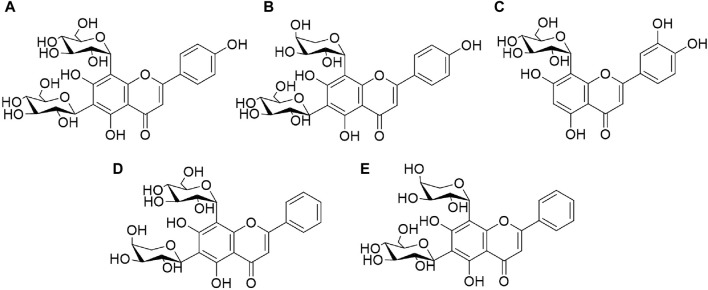
Chemical structures of five reference standards: vicenin-II **(A)**, orientin **(B)**, schaftoside **(C)**, chrysin 6-*C*-arabinoside 8-*C*-glucoside **(D)**, chrysin 6-*C*-glucoside 8-*C*-arabinoside **(E)**.

Reference standards of vicenin-Ⅱ (a), orientin (b), schaftoside (c), chrysin 6-*C*-arabinoside 8-*C*-glucoside (d), and chrysin 6-*C*-glucoside 8-*C*-arabinoside (e) were purchased from Chengdu Alpha Biotechnology Co. Ltd. (Chengdu, China). The structures of the five reference standards are shown in Figure 1. The estimated purities of all the standards were >95%, as determined with ultra-high performance liquid chromatography (UHPLC).

Acetonitrile (MS grade) was purchased from Fisher Scientific Co. (Loughborough, United Kingdom). Deionized water provided by a Millipore system (Millipore, Bedford, MA, USA) was used throughout the study. Formic acid (MS grade) was purchased from Acros Co. Ltd. (NJ, USA). Other reagents of analytical grade were obtained commercially from Sinopharm chemical reagent Co., Ltd. (Beijing, China).

### 2.3 Standard solutions and sample preparations

An accurately weighed amount of standard was dissolved in methanol to prepare the working solution. The concentrations of stock solution were 1.013, 0.398, 2.058, 1.988, and 1.964 mg/mL for compounds a, b, c, d, and e, respectively.

A proportion of each standard stock solution was further diluted with 70% ethanol aqueous solution to create an intermediate solution. The final concentrations of compounds a, b, c, d, and e, were calculated as 202.6, 179.5, 164.6, 119.3, and 157.1 μg/mL, respectively. A series of standard solutions with the desired concentrations (Table 1) were obtained by diluting the intermediate solution with 70% ethanol and utilized to construct calibration curves. Solution LIN100 (100% concentration) containing final concentrations of 50.66 μg/mL vicenin-Ⅱ, 44.87 μg/mL orientin, 41.15 μg/mL schaftoside, 29.82 μg/mL chrysin 6-*C*-arabinoside 8-*C*-glucoside, and 39.28 μg/mL chrysin 6-*C*-glucoside 8-*C*-arabinoside was selected as the standard solution.

**TABLE 1:** Different concentration levels of standard solutions (μg/mL).

Standard solution	Vicenin-II	Orientin	Schaftoside	Chrysin 6-*C*-arabinoside 8-*C*-glucoside	Chrysin 6-*C*-glucoside 8-*C*-arabinoside
LIN200	101.3	89.74	82.31	59.64	78.56
LIN150	75.99	67.31	61.73	44.73	58.92
LIN100	50.66	44.87	41.15	29.82	39.28
LIN50	25.33	22.44	20.58	14.91	19.64
LIN25	12.66	11.22	10.29	7.456	9.820
LIN10	5.066	4.487	4.115	2.982	3.928

The plant material was pulverized in a mortar and passed through a 0.28 mm mesh sieve. Fine sample powder (0.5 g) was accurately weighed, extracted with 30 mL of a mixture of ethanol and water (50:50/v:v), ultrasonic treatment for 20 min, and cooled to room temperature. After centrifugation at 9500 r/min for 20 min, the supernatant was passed through a 0.22 μm filter membrane prior to analysis.

### 2.4 Qualitative and quantitative analysis conditions

#### 2.4.1 Qualitative analysis via UHPLC-Q-TOF/MS

UHPLC-Q-TOF/MS analysis was performed on an ACQUITY UHPLC™ instrument (Waters Corp., Milford, MA, USA) coupled with a Vion IMS-Q-TOF system (Waters Corp., Wilmslow, United Kingdom). The analysis was performed using a Waters ACQUITY UHPLC HSS T3 column (100 × 2.1 mm, 1.8 μm), with the temperature set at 40°C. Water with 0.1% formic acid (A) and acetonitrile (B) was used as the mobile phase. The following gradient was employed: 0–15 min, 5%→50% B; 15–25 min, 50%→95% B; 25–27 min, 95%→95% B. The flow rate was 0.5 mL/min and injection volume of the sample was 2 μL.

The data acquisition mode was MS^E^. Data were obtained at 50–1500 Da. The source temperature was 110°C, desolvation temperature was 450°C with desolvation gas flow of 850 L/h, leucine enkephaline used as lock mass, and capillary voltage set to 3 kV (positive ion mode, ESI^+^) and 2.5 kV (negative ion mode, ESI^−^). In a low-collision energy (CE) scan, cone voltage was 30 V, while in a high CE scan, ramp CEs were 20–40 in the negative and positive ion modes. The data obtained were analyzed using UNIFI 1.9.4 software (Waters Corp. Milford, MA, USA).

#### 2.4.2 Quantitative analysis via UHPLC-DAD

Quantification of *L. hastata* samples was performed using a Thermo Scientific UltiMate 3000 UHPLC system (Thermo Fisher Scientific Inc., Waltham, USA). Separation was achieved using a Luna Omega Polar C18 100 Å column (Phenomenex, Torrance, California, USA; 1.6 µm, 2.1 × 100 mm). The mobile phase consisted of water containing 0.1% formic acid (A) and acetonitrile containing 0.1% formic acid (B). The gradient elution program was as follows: 0–42 min, 8%→12% B; 42–46 min, 12% B; 46–48 min, 12%→100% B; 48–58 min, 100% B; 58–60 min, 100%→8% B; 60–65 min, 8% B, with the column temperature, flow rate and injection volume set at 40°C, 0.5 mL/min and 1 μL, respectively. DAD detection was performed at a wavelength of 270 nm.

### 2.5 Validation of the quantitative method

Analytical parameters of the method were examined including system applicability, specificity, linearity, limit of detection (LOD), limit of quantification (LOQ), accuracy, precision (repeatability and intermediate precision), solution stability, and durability.

#### 2.5.1 System applicability, linearity, LOD, LOQ

A series of six concentrations (10%, 25%, 50%, 100%, 150%, and 200%) of the standard solutions, specifically, vicenin-Ⅱ (5.066–101.3 μg/mL), orientin (4.487–89.74 μg/mL), schaftoside (4.115–82.31 μg/mL), chrysin 6-*C*-arabinoside 8-*C*-glucoside (2.982–59.64 μg/mL), and chrysin 6-*C*-glucoside 8-*C*-arabinoside (3.928–78.56 μg/mL) (Table 1), were employed to plot linear calibration curves based on the peak area of chemical analytes (Y) *versus* the concentration of each analyte (X). LOD and LOQ values were individually determined by analyzing the reference standard solutions and calculated as analyte concentrations yielding signal-to-noise (S/N) ratios of approximately 3 and 10, respectively. In order to guarantee the system stability, six replicates of the standard solution LIN100 were injected continuously, the RSD of peak area reproducibility of targeted compound was used for evaluation of system applicability.

#### 2.5.2 Accuracy, precision (repeatability and intermediate precision), solution stability, and durability tests

To evaluate the accuracy of the developed method, recovery studies were performed. A specific amount of intermediate solution was spiked into known amounts of samples to desired concentration levels of 80%, 100%, and 120% for each standard. Three replicates for each concentration level were extracted and analyzed as described above. The recovery value (%) was calculated using the following equation: recovery (%) = 100 × (detected amount-original amount)/spiked amount.

Repeatability and inter-day precision were determined by analyzing six replicates at the middle concentration level of the standard solution within one and three consecutive days, respectively. Repeatability was verified by analyzing six replicates of the sample using the above method.

For assessment of stability, freshly prepared sample and standard solutions were stored at 10°C. Sample solutions were analyzed at 0, 3, 6, 9, 13, and 16 h, respectively, and standard solutions at 0, 5, 8, 11, 15, 18, and 21 h, respectively. Variations were expressed asrelative standard deviation.

To determine durability, the blank, standard, and duplicate sample solutions were analyzed under the above chromatographic conditions, with adjustments made to the flow rate (0.45 mL/min and 0.55 mL/min) and column temperature (38°C and 42°C), respectively.

All results were estimated by calculating the relative standard deviation (RSD) value. The corresponding acceptance criteria are clearly defined.

### 2.6 Quantitative analysis

Quantitative analysis of the sample was conducted using UHPLC-DAD. The quantities of the test product components were calculated from the slope of the curve according to the formula:

$$C=\frac{R_{u}‐a}{b},W=\frac{C\times V}{m}$$

Note: C, concentration of the component for measurement in sample solution, µg/mL; R_u_, peak area of the component to be measured in the test product solution; a, Standard curve intercept; b, Slope of the standard curve; W, content of components to be measured in sample solution, μg/g; V, Volume of sample solution, mL; m, quantity of sample solution, g.

## 3 Results and discussion

### 3.1 Identification of constituents of *Leptadenia hastata* via UHPLC-Q-TOF/MS

**FIGURE 2:**
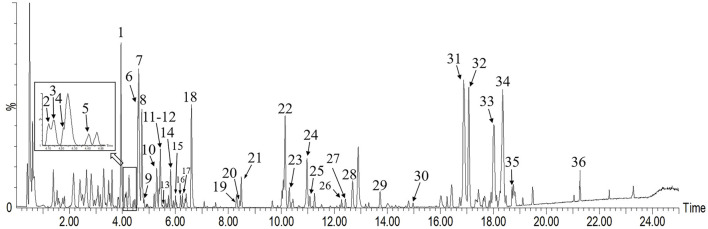
Base peak ion (BPI) chromatogram of *Leptadenia hastata* in the negative mode via UHPLC-Q-TOF/MS.

UHPLC-Q-TOF/MS analysis of the chemical constituents of *L. hastata* was conducted with meticulous optimization of MS parameters (encompassing ionization mode, capillary voltage, and various collision energy ranges). Herbal extract samples were examined in both positive and negative ion modes under identical LC conditions, with a significantly heightened base peak ion (BPI) response observed in the negative ion mode. All base peaks were identified by accurate molecular weight and ion fragments, with an error tolerance of 10 mDa. Tentative identification of 35 compounds (20 flavonoids and 15 organic acids) from the *L. hastata* extract was achieved, as presented in Table 2, five of which were validated with reference standards. The accuracy of this characterization was substantiated with data from high-resolution mass spectrometry, as illustrated in Figure 2. Integration of the measured molecular weights with fragment ion information acquired through collision-induced dissociation (CID) facilitated structural elucidation, revealing flavonoids and organic acids as the predominant constituents.

**TABLE 2:** Information on the main compounds in *Leptadenia hastata* extracts identified via UHPLC-Q-TOF/MS.

Peak No.	*t*_*R*_ (min)	Formula	Observed *m/z*	Adducts	Mass error (mDa)	Identification	Fragment ions
1	3.95	C_27_H_30_O_15_	593.1504	-H	−0.2	vicenin-II*	503.1195, 4731087, 413.0880, 383.0767, 353.0663
2	4.10	C_26_H_28_O_15_	579.1343	-H	−0.7	lucenin 1	519.1152, 489.1043, 459.0929, 399.0728, 367.1239
3	4.14	C_26_H_28_O_15_	579.1348	-H	−0.2	carlinoside	489.1059, 459.0932, 399.0729
4	4.21	C_28_H_32_O_16_	623.1606	+HCOO	−0.6	stellarin 2 or its isomer	503.1187, 383.0767
5	4.41	C_28_H_32_O_16_	623.1608	+HCOO	−0.4	stellarin 2 or its isomer	563.1414, 503.1207, 473.1091, 383.0771
6	4.59	C_26_H_28_O_14_	563.1395	-H	−0.6	schaftoside*	503.1200, 473.1085, 443.0514, 413.0902, 383.0768, 353.0662, 297.0405
7	4.61	C_21_H_20_O_11_	447.0924	-H	−0.3	orientin*	357.0609, 327.0504
8	4.73	C_21_H_20_O_11_	447.0925	+HCOO	−0.2	isomer of orientin	357.0610, 327.0504
9	4.82	C_26_H_28_O_14_	563.1403	-H	0.2	isomer of schaftoside	473.1089, 383.0760
10	5.29	C_27_H_30_O_16_	609.1453	-H	−0.3	rutin	301.0344, 283.0615
11	5.41	C_26_H_28_O_13_	547.1451	-H	−0.3	chrysin 6-*C*-arabinoside 8-*C*-glucoside*	487.1251, 457.1140, 427.1034、337.0715
12	5.43	C_27_H_30_O_15_	593.1503	-H	−0.1	luteolin 7-*O*-rutinoside or its isomer	285.0399
13	5.61	C_21_H_20_O_11_	447.0930	-H	0.3	Isomer of orientin	389.1611, 285.0400
14	5.82	C_26_H_28_O_13_	547.1450	-H	−0.2	chrysin 6-*C*-glucoside 8-*C*-arabinoside*	457.1136, 427.1026, 337.0707
15	6	C_27_H_30_O_15_	593.1499	-H	−0.7	luteolin 7-*O*-rutinoside or its isomer	285.0399
16	6.19	C_28_H_32_O_16_	623.1606	+HCOO	−0.6	stellarin 2 or its isomer	563.1364
17	6.26	C_28_H_32_O_16_	623.1603	+HCOO	−0.9	stellarin 2 or its isomer	503.2510
18	6.6	C_28_H_32_O_15_	607.1660	-H	−0.3	Chrysoeriol 7-O-neohesperidoside	299.0555, 284.0320
19	8.30	C_15_H_10_O_6_	285.0399	-H	0	luteolin	133.0307
20	8.43	C_26_H_30_O_13_	549.1606	-H	−0.2	liquiritigenin-7-*O*-*β*-D-apiofuranosyl-4′-*O*-*β*-D-glucopyranoside or its isomer	429.1176, 395.2119, 285.0408、255.0658
21	8.48	C_21_H_36_O_10_	493.2284	+HCOO	0	1,1′-(2,15-dihydroxytrimethyl-4,7,10,13-tetraoxahexadecane-1,16-diyl) ester or its isomer	343.1563, 315.1811, 162.8390
22	10.14	C_18_H_32_O_5_	327.2173	-H	0.2	9,12,13-Trihydroxy-10,15-octadecadienoic acid or its isomer	229.1440, 211.1335
23	10.3	C_18_H_32_O_5_	328.2173	-H	0.2	9,12,13-Trihydroxy-10,15-octadecadienoic acid or its isomer	229.1442, 211.1346
24	10.97	C_18_H_34_O_5_	329.2328	-H	0.2	tianshic acid or its isomer	229.1443, 211.1335
25	11.07	C_18_H_34_O_5_	329.2328	-H	−0.1	tianshic acid or its isomer	229.1445, 211.1331
26	12.27	C_18_H_30_O_4_	309.2065	-H	−0.2	decanediol dimethacrylate or its isomer	291.1976, 171.1043, 160.8419
27	12.42	C_18_H_30_O_4_	309.2065	-H	−0.2	decanediol dimethacrylate or its isomer	291.1976, 171.1043, 160.8419
28	12.68	C_18_H_28_O_4_	307.1908	-H	0	(8*E*,10*E*)-7,12-Dioxo-8,10-octadecadienoic acid	289.1809, 235.1340, 185.1182
29	13.72	C_18_H_28_O_4_	305.1751	-H	−0.1	(10*E*,12*Z*,14*E*)-9,16-Dioxo-10,12,14-octadecatrienoic acid	249.1496, 135.0810
30	14.97	C_18_H_30_O_4_	309.2065	-H	−0.2	decanediol dimethacrylate or its isomer	291.1976, 171.1043, 160.8419
31	16.88	C_18_H_30_O_3_	293.2114	-H	−0.3	9-Oxo-10*E*, 12*Z*-octadecadienoic acid or its isomer	275.2011, 235.1699
32	17.07	C_18_H_30_O_3_	293.2115	-H	−0.2	9-Oxo-10*E*, 12*Z*-octadecadienoic acid or its isomer	275.2014, 223.1336, 195.1385
33	18.01	C_18_H_32_O_3_	295.2271	-H	−0.2	coronaric acid	277.2169, 255.2326
34	18.35	—	555.2833	-H	—	Unknown	529.2991, 483.2735, 481.2605
							353.2008, 299.0433, 293.2127
							279.2347, 255.2324, 225.0074
							160.8419
35	18.7	C_18_H_30_O_3_	293.2117	-H	0	9-Oxo-10*E*, 12*Z*-octadecadienoic acid or its isomer	255.2330
36	21.24	C_18_H_30_O_2_	277.2167	-H	−0.1	linolenic Acid	225.2221

Note: * Identified compound validated by reference standards.

Flavone *C*-glycosides were characterized by a direct *C*-*C* bond linkage between the sugar moiety and flavonoid nucleus. The primary binding sites were at positions *C*-6 and *C*-8. In the negative ion mode, flavonoid glycosides undergo sequential loss of sugar moieties. Therefore, the type of sugar (pentose or hexose) could be deduced through high-energy mass spectrometry fragmentation (Ferreres et al., 2003). Neutral loss of C_3_H_6_O_3_ (90) and C_2_H_4_O_2_ is reported for pentoses (60) while cleavage of hexoses results in neutral loss of C_4_H_8_O_4_ (120) and C_3_H_6_O_3_ (90). This information was valuable for determining the sugar moiety associated with flavonoid glycosides during structural elucidation.

In the negative ion mode, peak six generated a quasi-molecular ion at *m/z* 563.1395 [M-H]^-^ and produced a series of deglycosylated fragments. The presence of *m/z* 503.1200 [M-H-C_2_H_4_O_2_]^-^ suggested the presence of a pentose in the structure while *m/z* 443.0514 [M-H-C_4_H_8_O_4_]^-^ indicated the existence of a hexose. Fragment ions at *m/z* 413.0902 [M-H-C_2_H_4_O_2_-C_3_H_6_O_3_]^-^, *m/z* 383.0768 [M-H-C_4_H_8_O_4_-C_2_H_4_O_2_]^-^, and *m/z* 353.0662 [M-H-C_4_H_8_O_4_-C_3_H_6_O_3_]^-^ were generated due to the neutral loss of sugar moieties, and the characteristic fragment *m/z* 297.0405 of the flavonoid nucleus was observed. Based on the relevant literature (Hou et al., 2023), peak six was identified as schaftoside. On the other hand, peak one did not produce [M-H-C_2_H_4_O_2_]^-^ fragment ions in the high CE scan and the remaining fragment ions were similar to peak 6. Therefore, the presence of two hexoses at positions *C*-6 and *C*-8 was inferred in the structure of peak 1. According to the component order of different glycosides in the C18 column chromatographic separation mode (Lai et al., 2016), peak one was identified as vicenin-Ⅱ.

**FIGURE 3:**
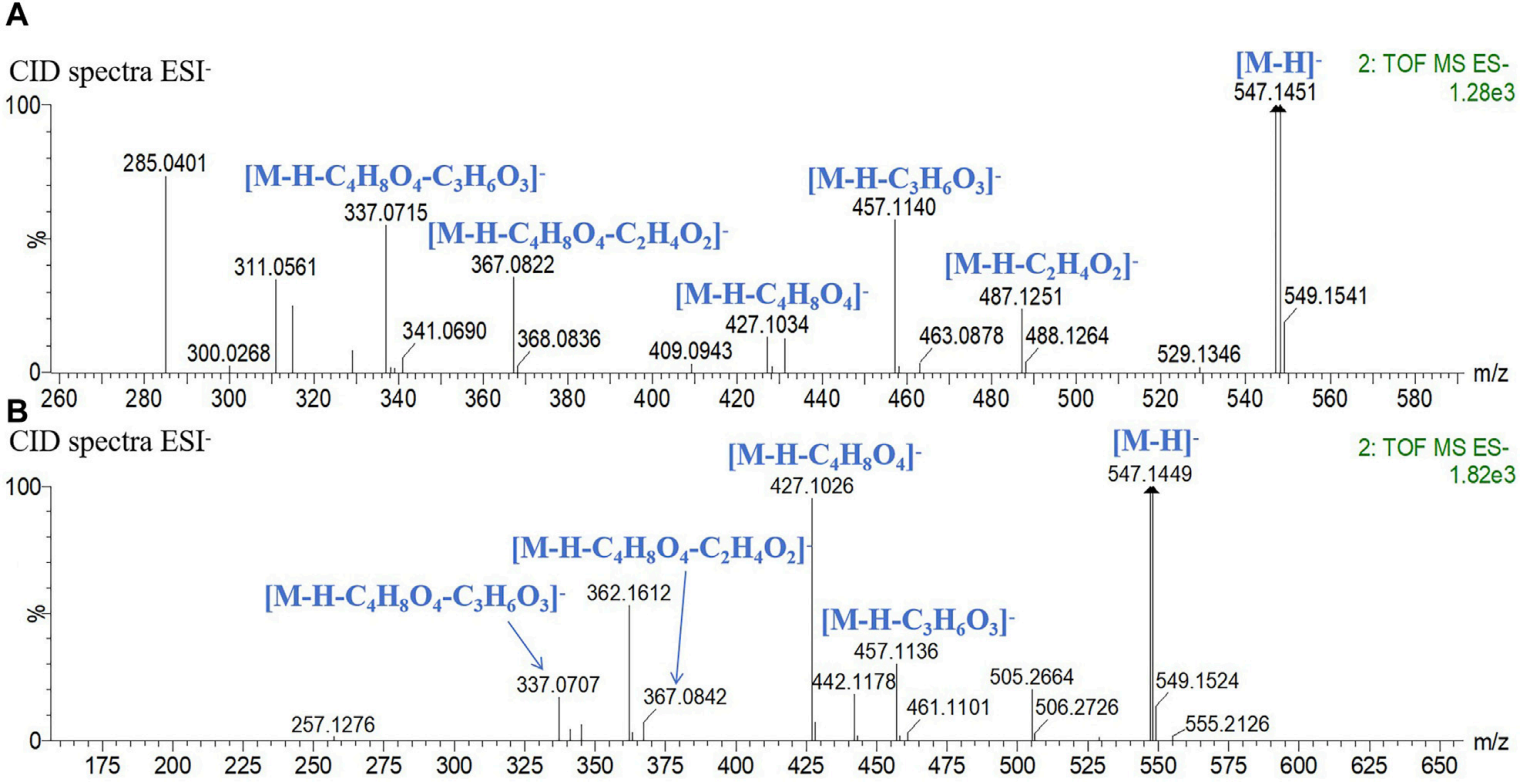
CID spectrum in the negative mode and fragment ion attribution. **(A)** chrysin 6-*C*-arabinoside 8-*C*-glucoside, **(B)** chrysin 6-*C*-glucoside 8-*C*-arabinoside.

Peaks 11 and 14 exhibited identical quasi-molecular ion peaks in the MS1 spectrum at *m/z* 547.1451 (C_26_H_27_O_13_, [M-H]^-^) and *m/z* 547.1450 (C_26_H_27_O_13_, [M-H]^-^). In the CID spectrum, characteristic fragment ions indicative of loss of C_2_H_4_O_2_ (60), C_3_H_6_O_3_ (90), and C_4_H_8_O_4_ (120) were observed, leading to the conclusion that both structures contain one molecule of hexose and one molecule of pentose. Fragment ions generated by peak 11 at *m/z* 487.1251 (C_24_H_22_O_11_, [M-H-C_2_H_4_O_2_]^-^) and *m/z* 457.1140 (C_23_H_21_O_10_, [M-H-C_3_H_6_O_3_]^-^) exhibited relatively higher abundance compared to *m/z* 427.1034 (C_22_H_19_O_9_, [M-H-C_4_H_8_O_4_]^-^), along with fragment ions produced by peak 14 at *m/z* 457.1136 (C_23_H_21_O_10_, [M-H-C_3_H_6_O_3_]^-^) and *m/z* 427.1026 (C_22_H_19_O_9_, [M-H-C_4_H_8_O_4_]^-^). The remaining fragment ions were identical for both peaks. The sugar moiety at the *C*-6 position is reported to be more prone to CID cleavage (Gao et al., 2022), as depicted in Figure 3. Accordingly, peak 11 was deduced to contain a pentose attached at *C*-6 while peak 14 had a hexose attached at *C*-6. Consequently, peaks 11 and 14 were identified as chrysin 6-*C*-arabinoside 8-*C*-glucoside and chrysin 6-*C*-glucoside 8-*C*-arabinoside, respectively.

The quasi-molecular ion peak with a retention time of 12.90 min in Figure 2 was observed at *m/z* 943.4874 (Supplementary Figure S1A). The chromatographic conditions of Supplementary Figure S1B were the initial settings, while the data presented in Supplementary Figure S1C was collected from blank solvent, and this ion peak was present in all chromatograms. This indicated that impurity peaks might be originated from blank solvent or analytical instrument, not from *L. hastata*. Peak 34 exhibited identical quasi-molecular ion peaks in the MS spectrum at *m/z* 555.2833 with a retention time of 18.35 min in Supplementary Figure S2A. Upon analyzing the fragments of the compound following CID cleavage in Supplementary Figure S2B, despite conducting searches in widely used mass spectrometry databases such as HMDB, PubChem, and Mass Bank, no suitable compound can be reasonably matched. Therefore, compound identification cannot be carried out temporarily.

### 3.2 Quantitative analysis of the five representative constituents in *Leptadenia hastata*

#### 3.2.1 Sample preparation

Three concentrations of methanol (30%, 50%, and 80% v/v) were assessed using samples SAM-1, SAM-2, and SAM-3. Extraction volumes of 10, 20, and 30 mL were compared across SAM-4, SAM-5, and SAM-6. Additionally, ultrasonic extraction times of 20, 30, and 40 min were evaluated with samples SAM-7, SAM-8, and SAM-9. As shown in Supplementary Figure S3, SAM-2 exhibited the highest peak area under a 50% methanol aqueous solution. Based on the similar and higher peak areas observed for SAM-5 and SAM-6 with 30 mL methanol, this volume was selected for further analysis. The extraction time of SAM-7 yielded the optimal extraction ratio for the majority of compounds at 20 min. Finally, samples SAM-10 and SAM-11 were subjected to 20 min of extraction with 30 mL of 50% ethanol, resulting in higher peak areas of analytes relative to other conditions. Thus, the optimal extraction method involved adding 30 mL of 50% ethanol solution, followed by ultrasonic extraction for 20 min.

#### 3.2.2 Optimization of UHPLC conditions

Chemical profiling of *L. hastata* disclosed the presence of numerous flavone C-glycosides, in particular, vicenin-Ⅱ (a), orientin (b), schaftoside (c), chrysin 6-*C*-arabinoside 8-*C*-glucoside (d), and chrysin 6-*C*-glucoside 8-*C*-arabinoside (e).

Initial screening of chromatographic columns revealed significant advantages of the Luna Omega Polar C18 100 Å column (Phenomenex, USA; 1.6 µm, 2.1 × 100 mm). Performance was further evaluated at a range of column temperatures (35, 40, 45, and 50°C). Our data showed that the five target components achieved satisfactory separation from adjacent components at 40°C.

Considering the lower specificity of the DAD detector compared to MS, combined with reference standards, the peak purity of each compound in the sample was examined at 190–400 nm. The peak shapes of the compounds in the reference standards were basically consistent with those of the corresponding compounds in the sample, confirming that each sample component was a single compound with no interference. Comparisons of the peak shapes of individual compounds from the reference standards and samples are shown in Supplementary Figure S4. Additionally, based on the spectral characteristics of the compounds, 270 nm was selected as the detection wavelength for quantitative analysis.

### 3.3 Method validation

#### 3.3.1 System applicability, linearity, LOD, and LOQ

All calibration curves for the five targeted analytes exhibited strong linearity, with correlation coefficients (*r* ^2^) exceeding 0.999 within the specified test ranges (Table 3). System applicability was validated, resulting in a range of RSD for peak areas of the five targeted compounds of 0.4%–0.6%, 0.3%–1.0%, 0.9%–1.6%, 0.6%–1.1%, and 0.3%–1.0%, respectively. All values were <5%, indicating that the system suitability and linearity of the method met acceptable standards. The limits of detection (LOD) and quantification (LOQ) for the compounds were 0.6–2.0 μg/mL and 1.8–3.6 μg/mL, respectively (Table 3). The chromatograms of five targeted compounds for LODs and LOQs are presented in Supplementary Figure S5.

**TABLE 3:** Summary of Linear regression, LODs, LOQs, precision, repeatability, stability and recovery for five representative compounds in *Leptadenia hastata*.

Compound	Linear regression			LOD^a^(μg/mL)	LOQ^b^(μg/mL)	Inter-day	Repeatability	Stability of sample solution	Stability of standard solution	Average recovery	
						(n = 6)	(n = 3)	(n = 6)	(n = 6)		
	Standard curve^c^	Linear range (μg/mL)	*r* ^2^			RSD (%)	RSD (%)	RSD (%)	RSD (%)	Mean (n = 3, %)	RSD (%)
vicenin-II	y = 0.0432x − 0.0126	5.019–100.4	1.0000	2.533	5.066	3.4	0.6	0.7	0.6	111.5	4.72
orientin	y = 0.0498x − 0.0545	4.481–89.61	0.9997	2.244	4.487	2.5	1.7	3.8	0.8	100.0	1.50
schaftoside	y = 0.0443x − 0.0201	4.074–81.47	0.9997	2.058	4.115	3.2	1.1	2.0	0.9	102.6	0.87
Chrysin 6-*C*-arabinoside 8-*C*-glucoside	y = 0.0610x − 0.0256	2.944–58.88	0.9998	1.491	2.982	3.2	1.0	2.4	0.6	104.4	3.79
chryin 6-*C*-glucoside 8-*C*-arabinoside	y = 0.0581x − 0.0197	3.992–79.84	0.9999	1.964	3.928	2.9	1.8	2.0	1.0	103.9	4.72

^a^ Limit of detection (S/N = 3).

^b^ Limit of quantification (S/N = 10).

^c^ Y: peak area of analytes; X: concentrations of standards (μg/mL).

#### 3.3.2 Accuracy, precision, repeatability, solution stability, and durability tests

The accuracy, precision (inter-day precision), repeatability, solution stability, and durability aspects of the method were further validated. The RSD values for inter-day precision (n = 6) of the five components were 2.5%–3.4% (Table 3) and RSD values for repeatability (n = 6) were 0.6%–1.8%, indicative of good precision and repeatability. Regarding accuracy, recovery values of the five analytes were with average recovery varying from 100.0% to 111.5%, and RSDs were no more than 4.72% (Table 3). Stability test results indicated that the sample solution remained stable at 10°C for up to 16 h, with RSDs of less than 3.8% (Table 3) for all five components of *L. hastata*. The standard solution was stable at 10°C for up to 21 h, with RSD of less than 1.0% (Table 3).

Based on results presented in Table 4, under the conditions of each adjusted method, the linear correlation coefficients (*r* ^2^) for the five targeted analytes ranged from 0.9887 to 1.0000 and the RSD range of peak area for all five compounds in standard solution was 0.1%–7.7%. Upon comparing the conditions of the normal and each adjusted method, the RSD range of the contents of the five compounds was determined as 1.6%–10.0%. In all four durability conditions, the results met the requirements. However, under the durability conditions of a flow rate of 0.45 mL/min and column temperature of 42°C, some impurities in the sample solution exhibited detection interference peaks with a separation degree of less than 1.0. Therefore, this method appears to be sensitive to both flow rate and column temperature, and regulation of the flow rate at 0.50–0.55 mL/min under chromatographic conditions along with a controlled column temperature of 38°C–40°C is recommended.

**TABLE 4:** Assessment of durability of the development method under different flow rate and column temperature conditions.

Standard	Linear correlation coefficient r^2a^				RSD of peak area^a^				RSD of content^b^			
	0.45^c^	0.55^c^	38^d^	42^d^	0.45^c^	0.55^c^	38^d^	42^d^	0.45^c^	0.55^c^	38^d^	42^d^
1	0.9890	0.9976	0.9999	0.9998	7.7	1.8	0.9	0.7	4.8	4.0	5.1	4.1
2	0.9904	0.9976	1.0000	0.9993	7.3	2.6	0.1	3.1	3.5	5.2	1.6	5.4
3	0.9887	0.9968	0.9997	0.9998	5.2	2.8	3.7	0.6	2.5	5.7	2.1	10.0
4	0.9895	0.9977	0.9997	0.9997	6.7	2.8	0.3	2.4	3.6	2.2	2.4	5.1
5	0.9898	0.9967	0.9999	0.9996	6.6	2.6	3.5	0.9	1.6	2.5	3.7	2.5

*Note*:^a^ Linear correlation coefficient of LH100 solution.

^a^ RSD, of peak area of LH100 Solution.

^b^ RSD, of content of sample solution.

^c^ Flow rate (mL/min).

^d^ Column temperature (°C).

Overall, the results were considered reasonable and acceptable, indicating that thisquantification method could be effectively utilized for evaluating the quality of *L. hastata.*

### 3.4 Quantitative analysis of samples via UPLC-DAD

The method developed in this study was applied to determine five target components in six batches of *L. hastata* samples. The representative chromatograms are presented in , Supplementary Figures S6–S8 and results are listed in Table 5. The quantities of the five flavonoid compounds in the plants were established in the order: orientin > vicenin-Ⅱ > chrysin 6-*C*-glucoside 8-*C*-arabinoside > chrysin 6-*C*-arabinoside 8-*C*-glucoside > schaftoside, with mean concentrations of 205.3 μg/g, 132.1 μg/g, 108.9 μg/g, 100.8 μg/g and 82.5 μg/g, respectively. In particular, accurate determination of the levels of orientin, vicenin-Ⅱ and schaftoside with known anti-inflammatory, antidiabetic, and hepatoprotective activities is critical (Islam et al., 2014; Liu et al., 2017; Nagai et al., 2018; Lee and Bae, 2019; Xiao et al., 2022). This facilitates the establishment of quality control measures for the plant based on its active constituents, enabling more comprehensive evaluation of its pharmacological activity and providing guidance for its clinical application.

**TABLE 5:** Contents of five targeted components in μg/g dry weight of six batches of plant material.

Component	Content (μg/g)						
	20211010	20211022	20211109	20220107	20220206	20220322	Average
Vicenin-II (1)	156.9	128.9	150.2	130.2	124.8	126.3	132.1
Orientin (2)	216.0	238.4	233.7	170.5	188.3	195.4	205.3
Schaftoside (3)	61.31	62.68	88.16	83.96	83.74	93.73	82.5
Chrysin 6-*C*-arabinoside 8-*C*-glucoside (4)	117.6	119.3	92.35	100.5	85.13	106.8	100.8
Chrysin 6-*C*-glucoside 8-*C*-arabinoside (5)	123.9	101.0	101.9	129.4	99.82	112.4	108.9

## 4 Conclusion

In the present study, a systematic and sensitive UHPLC-Q-TOF/MS method was established for the first time for qualitative analysis of chemical compounds in *L. hastata*. A total of 35 components derived from *L. hastata* were definitively identified or tentatively characterized, including 20 flavonoids and 15 organic acids. Among these, five representative constituents were employed as quantitative indicators to evaluate the quality of *L. hastata* using UHPLC-DAD based on their established pharmacological activities and contents. In summary, the chemical profiles of *L. hastata* were systematically characterized and the intrinsic quality of six batches of *L. hastata* samples assessed using a sensitive and reliable quantitative method. This study presents the development of a practical and effective holistic approach for regulation of the quality of *L. hastata*.

## Data Availability

The original contributions presented in the study are included in the article/Supplementary Material, further inquiries can be directed to the corresponding author.

## References

[B1] Abdallah B. M. Ali E. M. (2022). Therapeutic potential of green synthesized gold nanoparticles using extract of *Leptadenia hastata* against invasive pulmonary aspergillosis. J. Fungi 8, 442–458. 10.3390/jof8050442 PMC9146234 35628698

[B2] Aliero A. A. Wara S. H. (2009). Validating the medicinal potential of *Leptadenia hastata* . Afr. J. Pharm. Pharmacol. 3, 335–338. 10.5897/AJB2009.000-9071

[B3] Aquino R. Pizza C. De Tommasi N. De Simone F. (1995). New polyoxypregnane ester derivatives from *Leptadenia hastata* . J. Nat. Prod. 58, 672–679. 10.1021/np50119a004 7623046

[B4] Bayala B. Rubio-Pellicer M. T. Zongo M. Malpaux B. Sawadogo L. (2011). Anti-androgenic activity of Leptadenia hastata (Pers.) Decne: competitive effect of the aqueous extracts of the plant and the testosterone propionate on castrated immature rats. Biotechnol. Agron. Soc. Environ. 15, 223–229.

[B5] Bello O. M. Ibitoye T. Adetunji C. (2019). Assessing antimicrobial agents of Nigeria flora. J. King Saud. Univ.– Sci. 31, 1379–1383. 10.1016/j.jksus.2018.04.017

[B6] Bello O. M. Zaki A. A. Khan S. I. Fasinu P. S. Ali Z. Khan I. A. (2017). Assessment of selected medicinal plants indigenous to west Africa for antiprotozoal activity. Sou. Afr. J. Bot. 113, 200–211.

[B7] Chukwuma I. F. Nworah F. N. Apeh V. O. Omeje K. O. Nweze E. J. Asogwa C. D. (2022). Phytochemical characterization, functional nutrition, and anti-diabetic potentials of *Leptadenia hastata* (Pers.) Decne leaves: *in silico* and *in vitro* studies. Bioinf. Biol. Insights 16, 117793222211154–17. 10.1177/11779322221115436 PMC9379957 35982736

[B8] Ezike A. C. Ufere I. K. Akah P. A. Ezea S. C. Okoli C. O. (2016). Extracts of *Leptadenia hastata* leaf, a famine food and traditional remedy for furuncles, suppress inflammation in murine models. J. Diet. Suppl. 13 (13), 119–135. 10.3109/19390211.2015.1008609 25730529

[B9] Ferreres F. Silva B. M. Andrade P. B. Seabra R. M. Ferreira M. (2003). Approach to the study of C-glycosyl flavones by ion trap HPLC-PAD-ESI/MS/MS: application to seeds of quince (Cydonia oblonga). Phytochem. Anal. 14, 352–359. 14667061 10.1002/pca.727

[B10] Freiberger C. E. Vanderjagt D. J. Pastuszyn A. Glew R. S. Mounkaila G. Millson M. (1998). Nutrient content of the edible leaves of seven wild plants from Niger. Plant Foods Hum. Nutr. 53, 57–69. 10.1023/A:1008080508028 10890758

[B11] Galani B. R. T. Owona B. A. Chuisseu D. P. D. Machewere E. Ngantchouko C. B. N. Moundipa P. F. (2020). Hepatoprotective activity of *Leptadenia hastata* (Asclepiadaceae) on acetaminophen-induced toxicity in mice: *in vivo* study and characterization of bioactive compounds through molecular docking approaches. Biomed. Res. Int. 2020, 1–15. 10.1155/2020/3807234 PMC7482022 32953880

[B12] Gao W. Y. Li T. Zhou Y. Y. Li M. L. Wang L. N. Zhao H. Y. (2022). Qualitative and quantitative study on chemical constituents in scutellariae radix decoction. Chin. Trad. Herb. Drugs 53, 7339–7352. 10.7501/j.1ssn.0253-2670.2022.23

[B13] Hou B. Y. Zhang Z. Liu Y. Jia X. Yun L. Wang W. Q. (2023). Analysis of flavonoid constituents in the aerial parts of Glycyrrhiza in flata Batalin by UPLC- Q-Exactive Orbitrap-MS. J. Northwest. Pharm. 38, 1–14. 10.3969/j.issn.1004-2407.2023.06.001

[B14] Islam M. N. Ishita I. J. Jung H. A. Choi J. S. (2014). Vicenin 2 isolated from Artemisia capillaris exhibited potent anti-glycation properties. Food Chem. Toxicol. 69, 55–62. 10.1016/j.fct.2014.03.042 24713265

[B15] Lai L. C. Lin Y. Y. Chen F. L. Lai X. P. (2016). Analysis of main active components in desmodii styraciflii herba by HPLC-Q-TOF-MS and HPLC-DAD. Chin. Trad. Herb. Drugs 47, 3578–3585. 10.7501/i.issn.0253-2670.2016.20.006

[B16] Lee I. C. Bae J. S. (2019). Hepatoprotective effects of vicenin-2 and scolymoside through the modulation of inflammatory pathways. J. Nat. Med. 74, 90–97. 10.1007/s11418-019-01348-x 31350693

[B17] Liu M. J. Zhang G. H. Huang X. T. Chen H. Min J. B. Liu C. H. (2017). Protection effect of schaftoside on mice with non-alcoholic fatty liver induced by high fat diet. J. Trad. Chin. Med. 32, 5078–5081.

[B18] Manosroi J. Zaruwa M. Z. Manosroi A. (2011). Potent hypoglycemic effect of Nigerian anti-diabetic medicinal plants. J. Complement. Integr. Med. 8, 1–14. 10.2202/1553-3840.1482 22754948

[B19] Mathieu G. Meissa D. (2007). Traditional leafy vegetables in Senegal: diversity and medicinal uses. Afr. J. Tradit. Complement. Altern. Med. 4, 469–475. 10.4314/ajtcam.v4i4.31239 20161914 PMC2816516

[B20] Nagai S. Matsumoto C. Shibano M. Fujimori K. (2018). Suppression of fatty acid and triglyceride synthesis by the flavonoid orientin through decrease of C/EBP δ expression and inhibition of PI3K/Akt-FOXO1 signaling in adipocytes. Nutrients 130, 130–16. 10.3390/nu10020130 PMC5852706 29373533

[B21] Nikiéma J. B. Vanhaelen-Fastré R. Vanhaelen M. Fontaine J. De Graef C. D. Heenen M. (2001). Effects of antiinflammatory triterpenes isolated from Leptadenia hastata latex on keratinocyte proliferation. Phytother. Res. 15, 131–134. 10.1002/ptr.700 11268112

[B22] Oche A. M. O. Watson J. T. Hyedima G. S. Ufeli B. S. (2022). *Leptadenia hastata* leaf extract ameliorates oxidative stress and serum biochemical parameters in streptozotocin-induced diabetes in Wistar rats. J. Diabet. Metab. Disord. 21, 1273–1281. 10.1007/s40200-022-01017-z PMC9672294 36404850

[B23] Ojochegbe A. B. Adejoh I. P. Boniface M. T. Duniya S. V. Anna I. (2019). Activity of methanol extract of *Leptadenia hastata* leaves in alcohol-induced liver injury. Int. J. Adv. Multidiscip. Res. 6, 11–18. 10.22192/ijamr.2019.06.07.002

[B24] Sanda K. A. Sandabe U. K. Auwal M. S. Bulama I. Bashir T. M. Sanda F. A. (2013). Hypoglycemic and antidiabetic profile of the aqueous root extracts of *Leptadenia hastata* in albino rats. Pak. J. Biol. Sci. 16, 190–194. 10.3923/pjbs.2013.190.194 24171268

[B25] Sena L. P. Vanderjagt D. J. Rivera C. Tsin A. T. C. Muhamadu I. Mahamadou O. (1998). Analysis of nutritional components of eight famine foods of the Republic of Niger. Plant Foods Hum. Nutr. 52, 17–30. 10.1023/A:1008010009170 9839831

[B26] Xiao Q. F. Zhao Y. Ma L. Piao R. L. (2022). Orientin reverses acetaminophen-induced acute liver failure by inhibiting oxidative stress and mitochondrial dysfunction. J. Pharmacol. Sci. 149, 11–19. 10.1016/j.jphs.2022.01.012 35369900

