# Supplementary material for: Qualitative and quantitative chemical analysis of Leptadenia hastata: exploring a traditional african medicinal plant
Source: Front Chem. 2024 Apr 19;12:1397549. doi: 10.3389/fchem.2024.1397549 (PMC11066277; doi:10.3389/fchem.2024.1397549)
Supplement: Supplementary file 1 [file DataSheet1.docx]

Qualitative and Quantitative Chemical Analysis of *Leptadenia hastata*: Exploring a Traditional African Medicinal Plant

Jiangsheng Zhang^1^, Yi Nan^2^, Jie Su^1^, Aminu Usman Jibril^3^, Guiyuan Lv^1^*

^1^College of Pharmaceutical Sciences, Zhejiang Chinese Medical University, Hangzhou, P. R. China

^2^Graduate School, Tianjin University of Traditional Chinese Medicine, Tianjin, P. R. China

^3^Graduate School, Department of Computer Science, Bayero University, Kano, Nigeria

* **Correspondence:**

Guiyuan Lv

zjtcmlgy@163.com


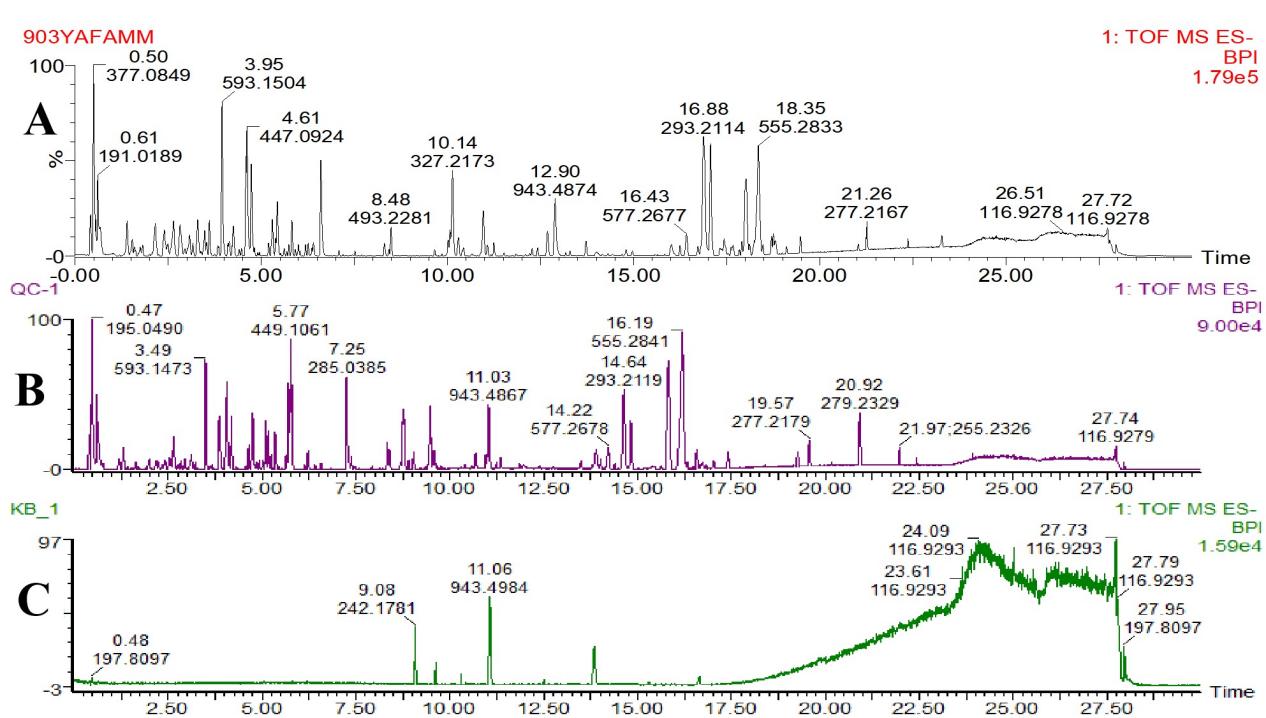


**Fig. S1.** BPI chromatograms in negative mode. (A) *L. hastata* extract with optimized condition. (B) *L. hastata* extract with initial condition. (C) Blank solvent with initial condition.


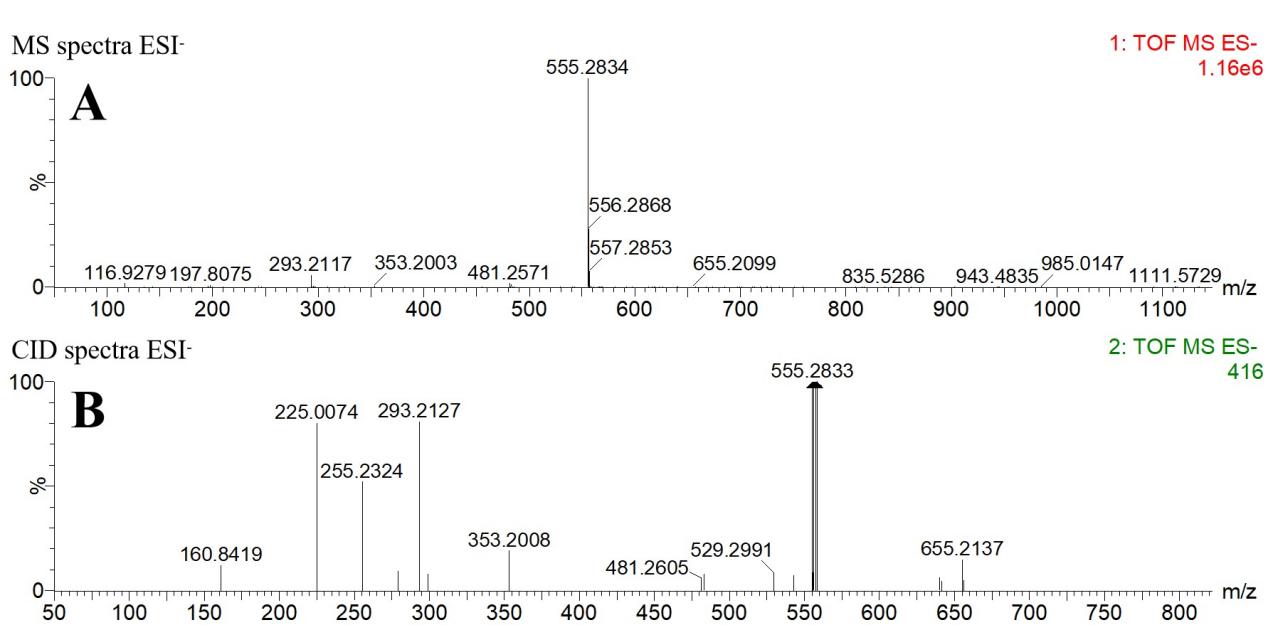


**Fig. S2.** Mass spectrograms in negative mode of base peak with retention time of 18.35 min (A) MS spectra. (B) CID spectra.

**Fig. S3.** Peak area of 5 target components in the sample solution made under different extract conditions.

a

b

c

d

e

f

g

h

i

j

**Fig. S4.** The peak purity of vicenin-Ⅱ（a, reference; b, sample）, orientin（c, reference; d, sample）, schaftoside（e, reference; f, sample）, chrysin 6-*C*-arabinoside 8-*C*-glucoside （g, reference; h, sample), chrysin 6-*C*-glucoside 8-*C*-arabinoside (i, reference; j, sample）


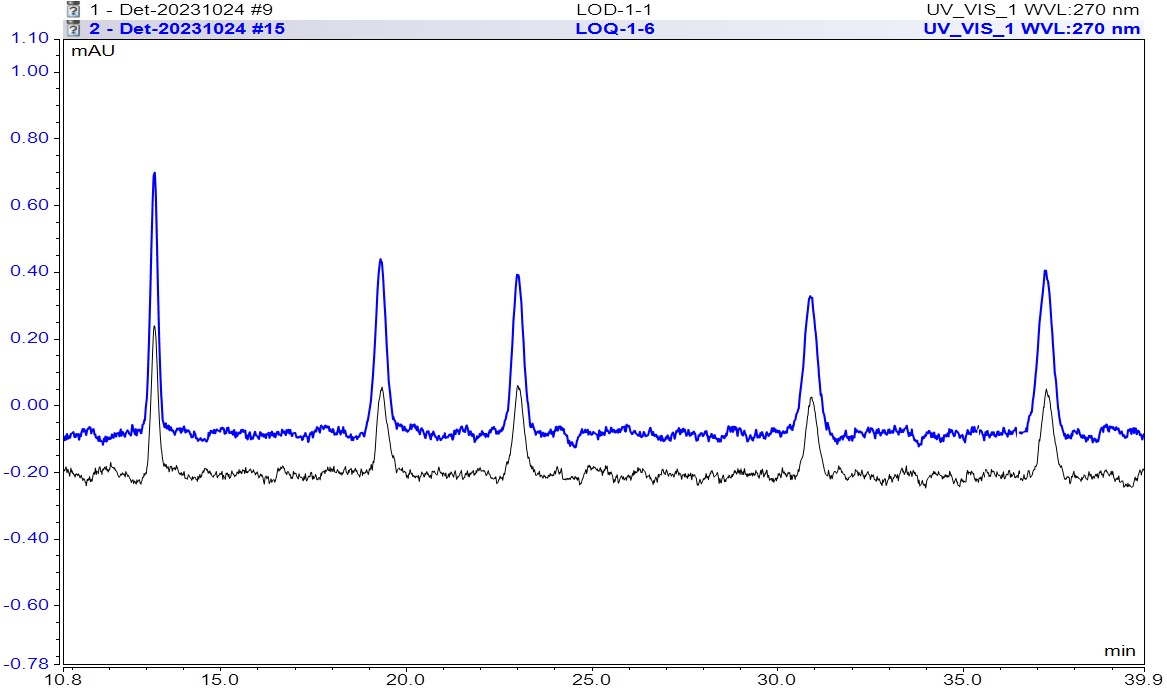


**a**

**b**

**c**

**d**

**e**

**Fig. S5.** The chromatograms of 5 targeted compounds for its LODs (black one) and LOQs (blue one).


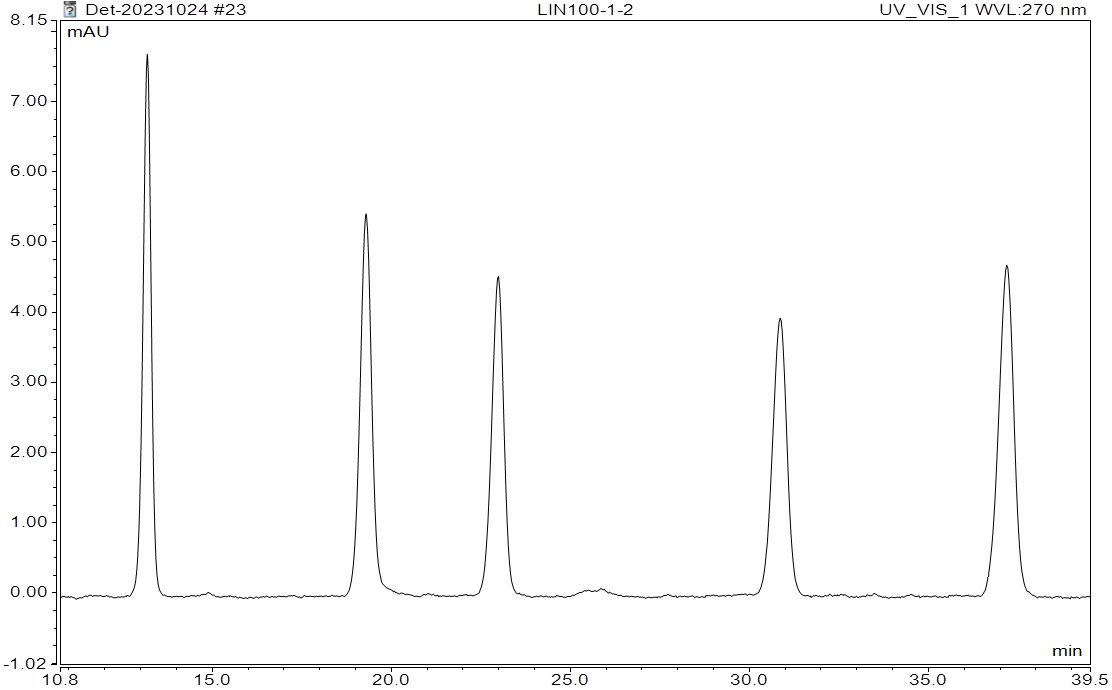


a

b

c

d

e

**Fig. S6.** The chromatograms of 5 mixed reference compounds in standard solution by UHPLC-DAD.


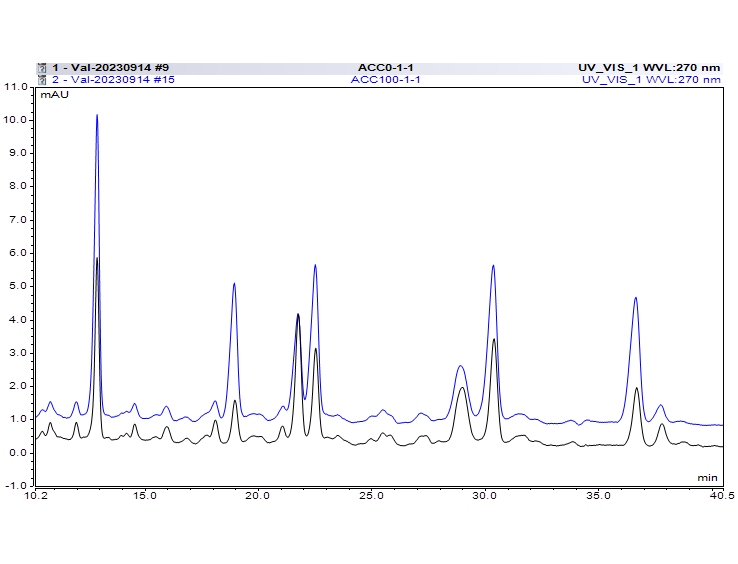


**a**

**b**

**c**

**d**

**e**

**Fig. S7.** The regionally magnified chromatograms of 5 compounds in *L. hastata* sample solution by UHPLC-DAD.


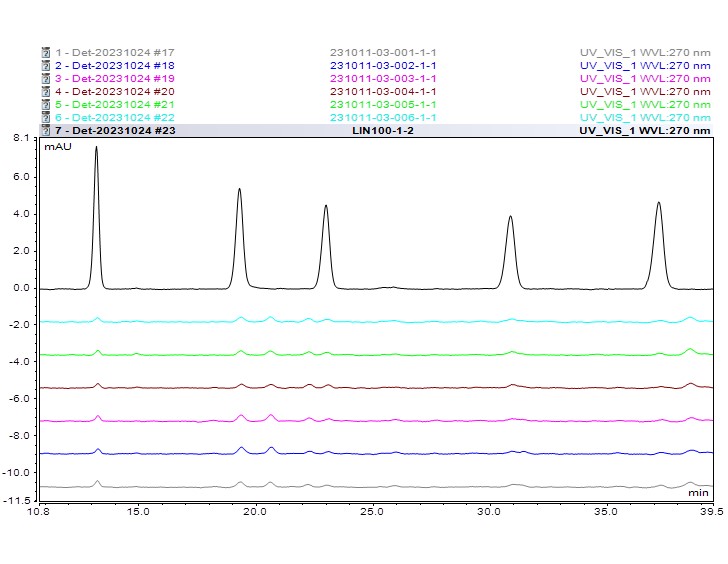


**a**

**b**

**c**

**d**

**e**

**Fig. S8.** The chromatograms of 5 targeted compounds in standard solution (LIN100) and 6 batch of sample solution by UHPLC-DAD.
